# 53BP1 depletion causes PARP inhibitor resistance in ATM-deficient breast cancer cells

**DOI:** 10.1186/s12885-016-2754-7

**Published:** 2016-09-09

**Authors:** Ruoxi Hong, Fei Ma, Weimin Zhang, Xiying Yu, Qing Li, Yang Luo, Changjun Zhu, Wei Jiang, Binghe Xu

**Affiliations:** 1 Department of Medical Oncology, Cancer Institute and Hospital, Peking Union Medical College and Chinese Academy of Medical Sciences, Beijing, China; 2 Department of Medical Oncology, Sun Yat-sen University Cancer Center, Guangzhou, China; 3 State Key Laboratory of Molecular Oncology, Cancer Institute and Hospital, Chinese Academy of Medical Sciences and Peking Union Medical College, Beijing, China; 4 College of Life Science/Tianjin Key Laboratory of Cyto-Genetical and Molecular Regulation, Tianjin Normal University, Tianjin, 300387 China

**Keywords:** 53BP1, PARP inhibitor, ATM, Drug resistance

## Abstract

**Background:**

Mutations in DNA damage response factors BRCA1 and BRCA2 confer sensitivity to poly(ADP-ribose) polymerase (PARP) inhibitors in breast and ovarian cancers. BRCA1/BRCA2-defective tumors can exhibit resistance to PARP inhibitors via multiple mechanisms, one of which involves loss of 53BP1. Deficiency in the DNA damage response factor ataxia-telangiectasia mutated (ATM) can also sensitize tumors to PARP inhibitors, raising the question of whether the presence or absence of 53BP1 can predict sensitivity of ATM-deficient breast cancer to these inhibitors.

**Methods:**

Cytotoxicity of PARP inhibitor and ATM inhibitor in breast cancer cell lines was assessed by MTS, colony formation and apoptosis assays. ShRNA lentiviral vectors were used to knockdown 53BP1 expression in breast cancer cell lines. Phospho-ATM and 53BP1 protein expressions were determined in human breast cancer tissues by immunohistochemistry (IHC).

**Results:**

We show that inhibiting ATM increased cytotoxicity of PARP inhibitor in triple-negative and non-triple-negative breast cancer cell lines, and depleting the cells of 53BP1 reduced this cytotoxicity. Inhibiting ATM abrogated homologous recombination induced by PARP inhibitor, and down-regulating 53BP1 partially reversed this effect. Further, overall survival was significantly better in triple-negative breast cancer patients with lower levels of phospho-ATM and tended to be better in patients with negative 53BP1.

**Conclusion:**

These results suggest that 53BP1 may be a predictor of PARP inhibitor resistance in patients with ATM-deficient tumors.

**Electronic supplementary material:**

The online version of this article (doi:10.1186/s12885-016-2754-7) contains supplementary material, which is available to authorized users.

## Background

Poly (ADP-ribose) polymerase (PARP) inhibitors have shown therapeutic potential in patients with ovarian and breast cancers associated with mutations in the breast and ovarian susceptibility genes *BRCA1* or *BRCA2* [1–3]. One randomized study in patients with relapsed high-grade serous ovarian cancer (HSOC) who had previously responded to platinum-based therapy found that progression-free survival (PFS) was significantly higher with the PARP inhibitor Olaparib (8.4 months) than with placebo (4.8 months; hazard ratio, 0.35; *P* < 0.001). Subset analysis showed greatest benefit in patients with germline or somatic mutations in *BRCA1* or *BRCA2,* in whom Olaparib prolonged PFS from 4.3 to 11.2 months (hazard ratio, 0.18; *P* < 0.001) [2]. These and other results led the US Food and Drug Administration to approve Olaparib for advanced ovarian cancer involving *BRCA* mutations, making it the first licensed PARP inhibitor drug.

PARP inhibitors compete with NAD^+^ binding, impairing the ability of PARP to produce PAR chains [4, 5]. Inhibition PARP-1 enzymatic activity results in the inability to recruit the appropriate DNA repair factors to the site of DNA damage, leading to SSB persistence, and these SSBs convert to DSBs, which are repaired by the error-free HR pathway at the replication fork [6]. Cells with defective BRCA1 or BRCA2 are unable to perform HR, so alternative repair processes kick in, such as non-homologous DNA end-joining (NHEJ). These alternative processes sometimes fail to repair DSBs, leading to genome instability and ultimately cytotoxicity. Consequently, cells deficient in BRCA1 or BRCA2 are highly sensitive to PARP-1 inhibition, which causes the accumulation of DSBs [6, 7]. PARP inhibitor therapy is based on synthetic lethality: it targets two separate molecular pathways that are non-lethal when disrupted independently, but are lethal when inhibited simultaneously [8]. Both BRCA1 and BRCA2 function in the homologous recombination (HR) pathway to repair of double-stranded DNA breaks (DSBs), while PARP-1 is a key mediator in the base excision repair (BER) pathway to repair single-stranded DNA breaks (SSBs) [9, 10].

Deficiency in several DNA damage response factors other than BRCA1 and BRCA2 have also been shown to be synthetically lethal with PARP inhibition [11, 12]. Screens based on short interfering RNA (siRNA) have identified several genes, such as ataxia-telangiectasia mutated (*ATM*), that when deleted sensitize tumor cells to PARP inhibitors [12]. ATM is a DNA damage-activated protein kinase and a member of the phosphatidyl-inositol kinase-like kinase (PIKK) family, which regulates responses to genotoxic stress, in particular DSBs [13]. In response to DNA damage, ATM autophosphorylates at Ser1981 and this activated form participates in cell cycle checkpoint arrest, DNA repair and/or apoptosis [13] by triggering phosphorylation of downstream effectors that include the checkpoint kinase Chk2, DNA repair factors such as BRCA1 and transcriptional regulators such as p53 [13, 14].

ATM alteration is most frequently seen in hematological cancers. For example, nearly 50 % of cases of mantle cell lymphoma contain mutations or deletions in *ATM* [15, 16]. ATM alteration is also common in solid tumors, including breast cancer, gastric and lung cancer [17]. Disrupting ATM, either through mutation, RNA interference or small-molecule inhibition, increase the sensitivity of cancer cells to PARP inhibitors [12, 18–20]. This suggests that PARP inhibitors may have therapeutic potential against ATM-deficient malignancies. It also raises the question of what additional genetic alterations may mediate or modulate synthetic lethality of PARP and ATM inhibition.

A candidate genetic event that may affect this synthetic lethality is loss of the DNA damage response factor 53BP1. So-called because it was first identified as a p53-binding protein, 53BP1 participates in both HR and NHEJ. 53BP1 stimulates NHEJ, whereas BRCA1 promotes end resection and HR [21–23]. Loss of 53BP1 appears to render BRCA1/BRCA2-defective tumors resistant to PARP inhibitors [21, 22], and studies *in vitro* and *in vivo* suggest this is because loss of 53BP1 partially restores the impaired HR in BRCA1-deficient cells [22]. This helps protect the genome and reduces the cytotoxicity of PARP inhibitors and DNA-damaging agents. Several BRCA1-deficient mouse mammary tumors that initially responded to Olaparib and later became resistant were shown to have lost 53BP1 and partially recovered HR [24].

Here we examined whether ATM inhibition may sensitize breast cancer lines to PARP inhibitors, as well as whether the functional status of 53BP1 may affect the sensitivity of ATM-deficient tumors to these inhibitors. We show that ATM inhibition enhanced the sensitivity of triple-negative and non-triple-negative breast cancer cell lines to Olaparib, and 53BP1 knock-down partially reversed this effect. ATM inhibition impaired HR induced by PARP inhibitor, and 53BP1 down-regulation partially restored HR. These results suggest that PARP inhibitors may be therapeutically useful against ATM-deficient breast cancer, and that the presence or absence of 53BP1 may predict which ATM-deficient tumors are likely to respond to such therapy.

## Methods

### Cell culture

Breast cancer cell lines CAL-51 and ZR-75-1 were cultured in RPMI 1640 (Gibco) with 10 % FBS. Cell lines MCF-7, T47D, MDA-MB-231, MDA-MB-468 and SK-BR-3 were cultured in DMEM (Gibco) containing 10 % FBS. All cultures were maintained at 37 °C in a 5 % CO_2_ atmosphere.

### Lentiviral transfection of target cells

Lentivirus encoding the short hairpin RNAs shRNA1 and shRNA2 targeting 53BP1 and the non-specific target shRNA were designed and prepared by Genechem (Shanghai, China). CAL-51 and MCF-7 cells were infected with a pool of lentiviruses carrying shRNA1 or shRNA2 and selected with 1 μg/mL puromycin for 10 days.

### Inhibitor treatment

The PARP-1 inhibitor Olaparib (AZD2281) and ATM inhibitor KU55933 were purchased from Selleck Chemicals. Compounds were serially diluted in DMSO and finally diluted 10^3^-fold in culture medium immediately before use.

### Cell proliferation assay

Cells were seeded in 96-well plates at the appropriate density for each cell line in 100 μl of medium and left at room temperature for 30 min, after which they were incubated overnight at 37 °C to allow attachment. Then cells were incubated for 48 h at 37 °C with Olaparib at concentrations of 0, 1, 2.5, 5, or 10 μM in the presence or absence of 10 μM KU55933. During incubation with inhibitors, the medium was not changed, nor were inhibitors added again.

Then 10 μl of MTS reagent (Promega, USA) was added directly to the wells, and plates were incubated at 37 °C for at least 1 h. Absorbance was measured at 490 nm on a microplate reader (Bio-Rad 680, USA). Background absorbance was first subtracted using a set of wells containing medium only, then normalized to and expressed as a relative percentage of the plate-averaged DMSO control.

### Colony formation assay

Cells (5 x 10^3^) were seeded into 6-well culture plates and incubated for 10 days at 37 °C in the presence of Olaparib at 0, 2.5, 5, or 10 μM in the presence or absence of 10 μM KU55933. Cells were washed with pre-chilled phosphate-buffered saline, fixed for 20 min with pre-chilled methanol and stained for 15 min with crystal violet. Colonies were examined and automatically counted using G:box (SYNGENE). All experiments were performed in triplicate.

### Flow cytometry-based apoptosis assay

Human breast cancer cell lines CAL-51 and MCF-7 were treated for 48 h with DMSO vehicle, 10 μM KU55933, 10 μM Olaparib, or the combination of 10 μM Olaparib and 10 μM KU55933. Cells were harvested by trypsinization, resuspended to a density of 1 × 10^6^ cells/mL in 1 × binding buffer, and stained with FITC-Annexin V and propidium iodide (PI) using the FITC Annexin V Apoptosis Detection Kit I (BD Biosciences). Stained cells were analyzed using an LSRII flow cytometer (Falcon BD, San Jose, CA, United States) according to the manufacturer’s instructions. All experiments were performed in triplicate.

### Western blotting

Cells were harvested and lysed on ice for 40 min in RIPA buffer (10 mM Tris pH 7.4, 150 mM NaCl, 1 % Triton X-100, 0.1 % Na-deoxycholate, 0.1 % SDS and 5 mM EDTA) containing Complete Protease Inhibitor Cocktail (Roche Applied Science). Total protein concentration was determined using a colorimetric assay. Equal amounts of total protein (60 μg) were separated by 6 %, 8 %, or 10 % SDS-PAGE and transferred to PVDF membranes. Membranes were blocked with 2 % bovine serum albumin (BSA), then incubated with primary antibodies overnight at 4 °C. Primary antibodies from Cell Signaling (Boston, MA, USA) were against caspase 3, cleaved caspase 3, caspase 8, cleaved caspase 8, caspase 9, cleaved caspase 9, PARP-1, cleaved PARP-1, Chk2, phospho-Chk2 (Thr68), phospho-ATM (Ser1981) and γ-H2AX. Primary antibodies from Abcam were against ATM and Rad51. Primary antibody against β-actin (Sigma) provided a loading control. The membrane was then incubated for 1 h with horseradish peroxidase-conjugated goat anti-mouse IgG (1:2000) or goat anti-rabbit IgG (1:3000) secondary antibody. The membrane was rinsed in 1 × PBS containing 0.1 % Tween, then incubated with chemiluminescence substrate. Blots were photographed using an Image Reader LAS-4000 (Fujifilm) and analyzed using Multi Gauge 3.2.

### Nuclear morphology assay

CAL-51 and MCF-7 cells were treated with DMSO, 10 μM Olaparib with or without 10 μM KU55933 respectively for 48 h, washed three times with PBS, fixed with methanol, and permeabilized with 0.1 % Triton X-100. Then cells were stained with DAPI at 37 °C for 5 min in the dark. Stained cells were washed twice with PBS before observed under fluorescence microscopy.

### γ-H2AX focus formation assay

CAL-51 and MCF-7 cells were treated for 48 h with DMSO vehicle or with Olaparib (5 or 10 μM) in the presence or absence of 10 μM KU55933, then fixed with 4 % paraformaldehyde. Fixed cells were incubated for 1 h in 1 % BSA/10 % normal goat serum/0.3 M glycine containing 0.05 % PBS-Tween in order to permeabilize the cells and block non-specific protein–protein interactions. Cells were incubated with anti-γ-H2AX antibody (Cell Signaling), followed by Alexa Fluor® 488-conjugated secondary antibody (ZSGB-BIO, Beijing, China). Nuclei were stained using DAPI. Cells were visualized using a non-confocal Operetta automated microscope (Perkin Elmer). The number of γ-H2AX foci per cell was quantified by ImageJ software. At least 200 cells per experiment point were examined.

### Clinical specimens

Formalin-fixed, paraffin-embedded (FFPE) tumor samples were obtained from breast cancer patients treated at the Cancer Institute and Hospital of the Chinese Academy of Medical Sciences and Peking Union Medical College. Clinical and follow-up data on the patients were extracted from the clinical database. The use of clinical specimens and data was approved by the local ethics committee of the Cancer Institute and Hospital of Peking Union Medical College.

### Immunohistochemistry of clinical specimens

FFPE samples of triple-negative breast cancer were immunohistochemically stained with a monoclonal antibody against phospho-ATM (1:100; rabbit IgG; Epitomics, clone EP1890Y, S1981). This antibody binds specifically to ATM phosphorylated Ser1981 [25]. Samples were stained for 53BP1 using a rabbit polyclonal antibody against 53BP1 (1:100; rabbit IgG; Santa Cruz Biotechnology, clone H-300, catalog no. SC-22760). Slides were deparaffinized with xylene and rehydrated in graded ethanol. Sections were submerged in EDTA antigenic retrieval buffer (pH 8.0), microwaved to retrieve antigens, treated with 3 % hydrogen peroxide in methanol to quench endogenous peroxidase activity, and finally incubated with 1 % goat serum albumin to block non-specific binding. Sections were incubated overnight at 4 °C with antibodies against phospho-ATM or 53BP1, washed, then treated for 40 min with goat anti-mouse or -rabbit IgG conjugated to horseradish peroxidase (ZSGB-BIO). The chromogen was 3,3′-diaminobenzidine.

### Semi-quantitation of immunohistochemical results

Intensity of staining for phospho-ATM and 53BP1 was scored as negative (0), weak positive (1), positive (2), and strong positive (3). In addition, the proportion of cells with each intensity score was recorded for each tissue section. We defined phospho-ATM immunostaining as ‘*high’* if at least 50 % of tumor cells had a nuclear staining intensity of at least 2. 53BP1 expression was defined as negative if more than 90 % of tumor cells showed negative nuclear staining (0). Immunostaining was semi-quantitated in this way by two observers working independently and blinded to clinical data. Inter-observer agreement was >90 %. Discrepancies were re-examined to obtain a consensus result.

### Statistical analysis

Statistical analysis was carried out using SPSS 20 (IBM, Chicago, IL, USA) or GraphPad Prism 5 for Windows, with a significance threshold of *p* < 0.05. Results were expressed as mean ± SEM, and differences were assessed for significance using the two-tailed Student’s t test. The two-tailed Pearson χ^2^ test or Fisher’s exact test was used to identify associations of phospho-ATM or 53BP1 expression with clinicopathological parameters. Survival curves were plotted using Kaplan-Meier analysis and compared using the log-rank test. Factors associated with survival were identified using multivariate Cox regression.

## Results

### ATM inhibition sensitizes breast cancer cells to Olaparib

**Fig. 1:**
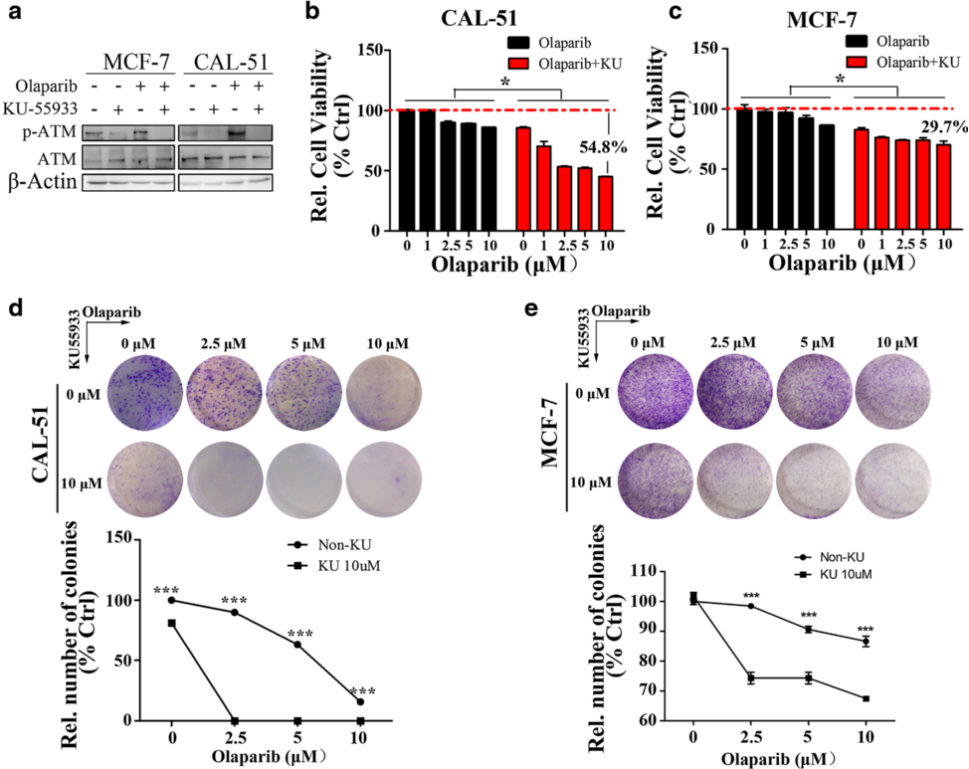
ATM inhibitor KU55933 sensitizes breast cancer cells to Olaparib **a**. Western Blot examined the protein level of phospho-ATM (Ser1981), ATM in MCF-7 and CAL-51 cells 48 h after treatment of 10 μM Olaparib, 10 μM KU55933 or their combination, respectively. **b**, **c** MTS assay was used to determine cell viability of CAL-51 and MCF-7 cells under different concentration of compounds treatments (0, 1, 2.5, 5, 10 μM Olaparib with or without 10 μM KU55933, respectively) at 37 °C for 48 h. * means that there is statistical difference between the groups of Olaparib treatment with or without KU55933 at each indicating concentration. **d**, **e**, Representative pictures (*Up*) and quantitative analysis (*bottom*) of colony formation assays. All experiments were performed at least three times and data were statistically analyzed by two-tail t-test.*p < 0.05, ***p < 0.001. Error bars indicate S.E.M

To evaluate whether ATM inhibition increases the cytotoxicity of PARP inhibitors, we treated both triple-negative breast cancer cell lines (CAL-51, MDA-MB-231 and MDA-MB-468) and non-triple-negative breast cancer cell lines (MCF-7, T-47D and SK-BR-3) with increasing concentrations of Olaparib (0–10 μM) alone or in combination with 10 μM ATM inhibitor KU55933. All cell lines had wild-type *BRCA1* and *BRCA2* genes; Additional file 1: Table S1 summarizes the ER, PR, HER2, BRCA1 and BRCA2 status of the lines. After 48-h incubation with one or both inhibitors, cell viability was assessed using the MTS assay. Olaparib induced ATM phosphorylation at Ser1981, which was substantially inhibited in the presence of KU55933 (Fig. 1a). This suggests that ATM activation participates in the cellular response to PARP inhibition. KU55933 significantly increased Olaparib toxicity in the triple-negative cell line CAL-51 and non-triple-negative cell line MCF-7 (Fig. 1b-c). Similar results were observed in the other cell lines (Additional file 1: Figure S1A). Interestingly, the three triple-negative cell lines appeared to be more sensitive to Olaparib in the presence of KU55933 than the three non-triple-negative cell lines. Olaparib inhibited colony formation in CAL-51 and MCF-7 cells in a dose-dependent manner, which KU55933 further enhanced it (Fig. 1d-e). Taken together, these data suggest that Olaparib suppresses cell proliferation and colony formation in breast cancer cell lines, and that KU55933 reinforces this effect through synthetic lethality.

### Treatment with Olarparib, KU-55933 or both induced formation of γ-H2AX foci

**Fig. 2:**
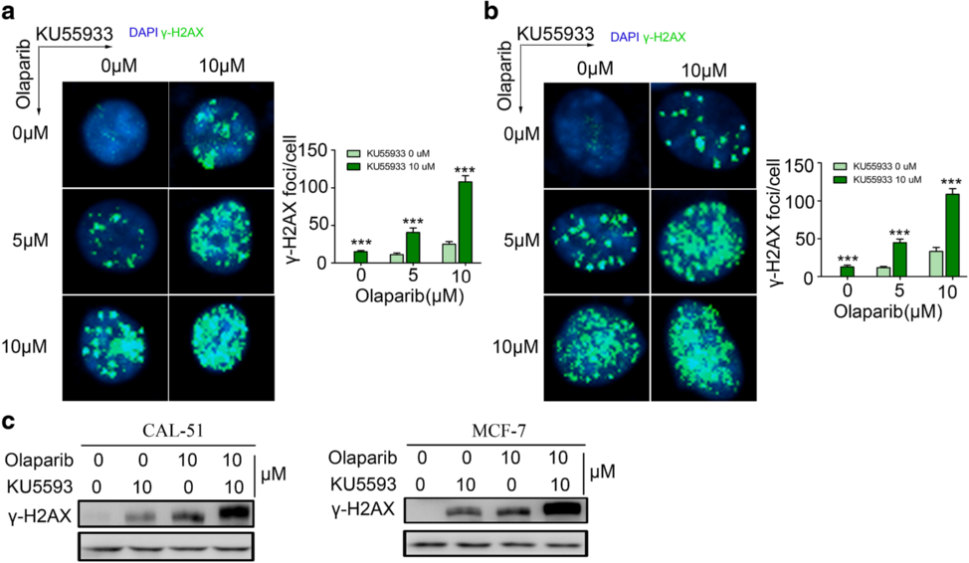
Treatment of Olarparib, Ku-55933 or their combination induced γ-H2AX foci formation A, B, CAL-51 cells (**a**) and MCF-7 cells (**b**) were treated with 0, 5, 10 μM Olaparib with or wihout 10 μM KU55933 respectively for 48 h and analyzed by immunofluorescence (IF) with γH2AX antibody. DAPI was used to visualize cell nuclei. Representative pictures (*left*) and quantitative data (*right*) of the γ-H2AX foci. **c**, CAL-51 cells (*left*) and MCF-7 cells (*right*) were treated with 0, 10 μM Olaparib with or wihout 10 μM KU55933 respectively 48 h and analyzed by Western Blot assay (WB) with γH2AX antibody. All experiments were performed at least three times and data were statistically analyzed by two-tail t-test. ***p < 0.001. Error bars indicate S.E.M

An early cellular response to DSBs is rapid phosphorylation of H2AX at Ser139, forming γ-H2AX foci [26]. H2AX phosphorylation plays a key role in signalling and initiating the repair of DSBs, and the numbers of γ-H2AX foci correlate closely with the number of DSBs [27]. Treating breast cancer cell lines with both Olaparib and KU55933 led to significantly more γ-H2AX foci formation than treating them with either inhibitor alone based on fluorescence microscopy (Fig. 2a-b). These findings were further confirmed by Western Blot assay (Fig. 2c). This provides further evidence that that Olaparib and Ku-55933 synthetically induce cellular DSBs.

### ATM inhibition enhanced Olaparib-induced apoptosis in MCF-7 and CAL-51 cells

**Fig. 3:**
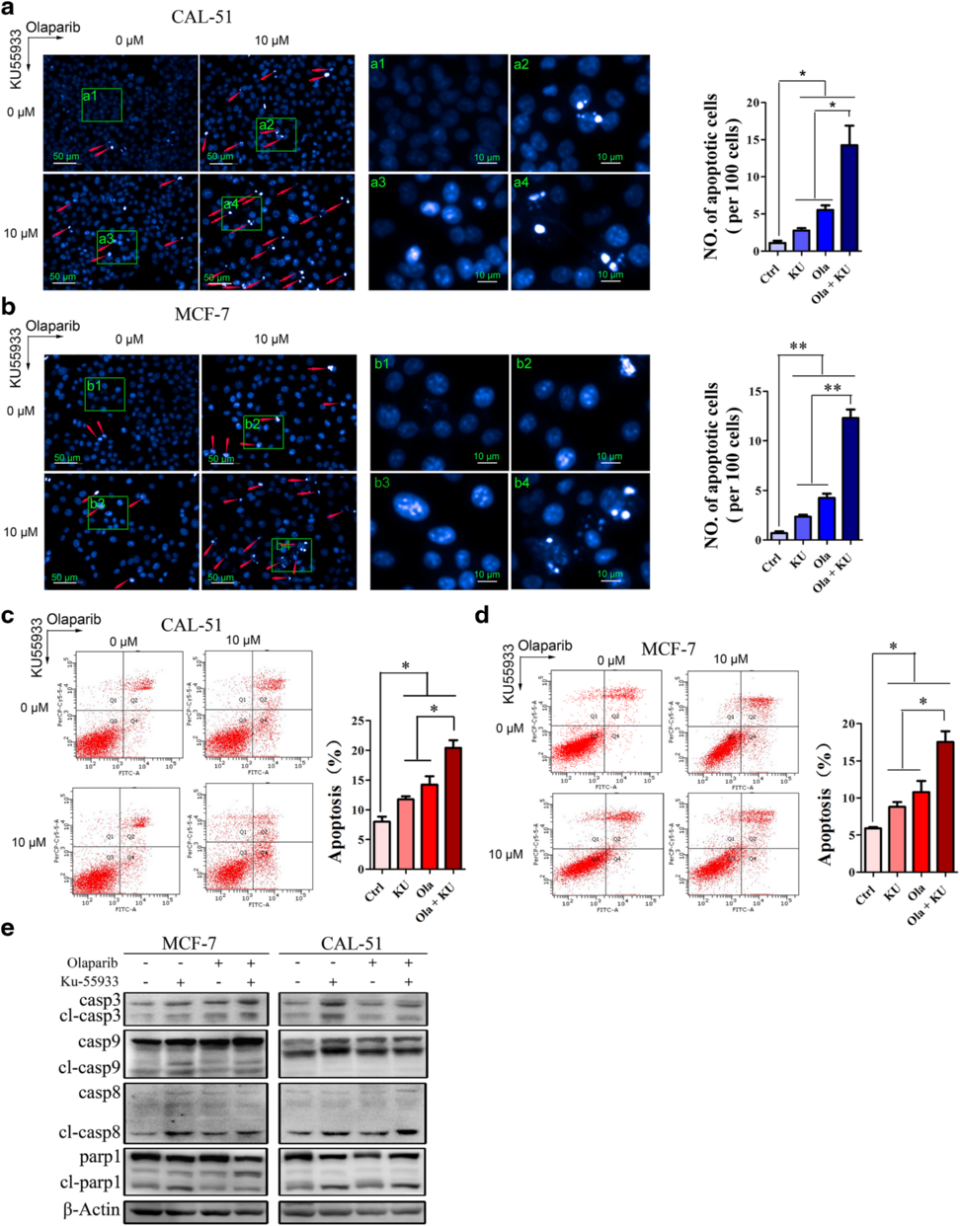
ATM inhibition enhanced Olaparib induced apoptosis in MCF-7 and CAL-51 cells **a**, **b**, DAPI staining indicated the nuclear morphological changes of CAL-51 cells (**a**) and MCF-7 cells (**b**). Cells were treated with 0, 10 μM Olaparib with or wihout 10 μM KU55933 respectively for 48 h. Left, representative pictures; right, quantitative data. **c**, **d**, Annexin V/PI staining and FACS analysis revealed cell apoptosis of CAL-51 cells (**c**) and MCF-7 cells (**d**). Cells were treated with 0, 10 μM Olaparib with or wihout 10 μM KU55933 respectively for 48 h. Left, representative pictures; right, quantitative data. **e**, Western Blots showed that caspase 3, caspase 8, caspase 9 and their cleaved forms were increased after treatment with 10 μM Olaparib or 10 μM KU55933 or their combination in CAL-51 cells and MCF-7 cells. All experiments were performed at least three times and data were statistically analyzed by two-tail t-test.*p < 0.05, **p < 0.01. Error bars indicate S.E.M

Since the experiments above indicated that Olaparib and KU55933 increased DSB formation, we wanted to know whether they induced changes in nuclear morphology. Such changes could reflect DSB-induced chromosome rearrangements or mutations, which can lead to apoptosis or death [23]. Treating MCF-7 and CAL-51 cells for 48 h with 10 μM Olaparib, 10 μM KU55933 or both led to nuclei with chromatin condensation and apoptotic bodies detectable by DAPI fluorescence (Fig. 3a-b), with the combination treatment causing more severe changes in nuclear morphology than either inhibitor alone. Similarly, combination treatment caused significantly greater apoptosis than Olaparib alone, based on Annexin V/PI staining followed by fluorescence-activated cell sorting (Fig. 3c-d). In addition, Western blotting experiments showed that combination treatment, as well as KU55933 treatment on its own, up-regulated expression of apoptotic caspases 3, 8, and 9, and their cleaved forms and it stimulated PARP-1 cleavage (Fig. 3e). These results suggest that Olarparib and KU55933 activate apoptosis pathways.

### 53BP1 knock-down reduced the hypersensitivity of ATM-inhibited cells to PARP inhibitor

Loss of 53BP1 reverses the hypersensitivity of *BRCA1*-deleted cells to the DNA cross-linker cisplatin by partially restoring HR and thereby protecting the genome. ATM, like BRCA1, plays a critical role in the DNA damage response. ATM kinase is activated following PARP inhibition and is required for PARP inhibitor induced HR. Thus, we asked whether 53BP1 down-regulation might render ATM-inhibited cells less sensitive to Olaparib.

**Fig. 4:**
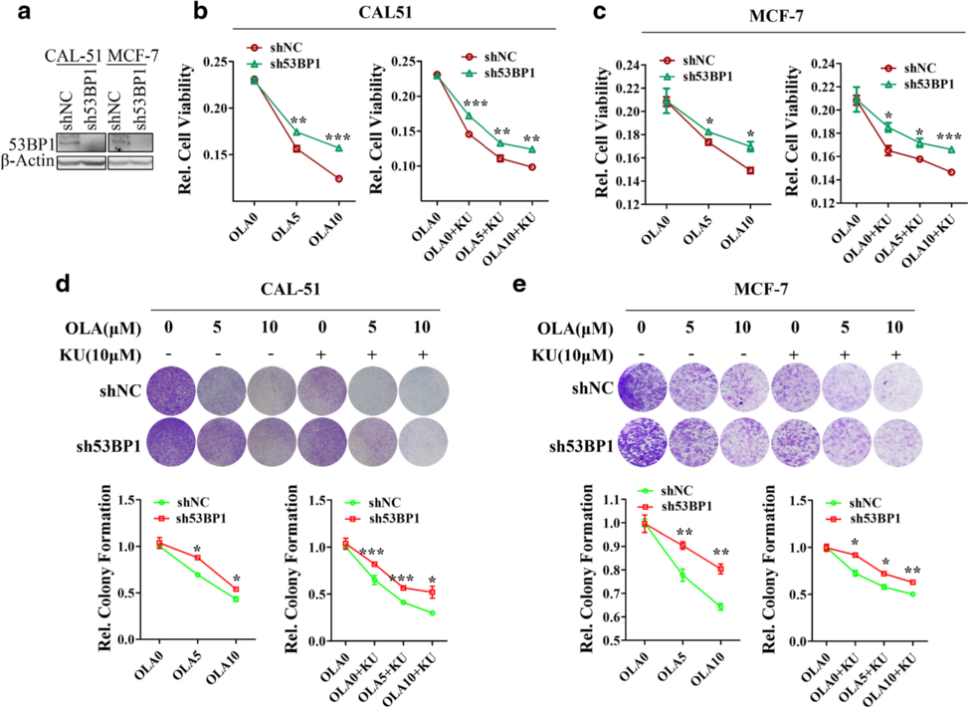
Knock down of 53BP1 reduces the hypersensitivity of PARP inhibitor in ATM inhibited cells **a**, Western blot shown that 53BP1 was efficiently knocked down by 53BP1 shRNA pool in CAL-51 cells and MCF-7 cells. **b**, **c**, MTS assays revealed the cell viability after treatment with indicated compounds for 48 h in CAL-51-sh53BP1 cells and their control counterparts (**b**) or in MCF-7-sh53BP1 cells and control-transfected cells (**c**). Left, cells were treated with 0, 5, 10 μM Olaparib. Right, cells were treated with 0, 5, 10 μM Olaparib combined with 10 μM KU55933. Cell viability was normalized to the 0 μM Olaparib (DMSO) treatment group. **d**, **e**, Colony formation assays showed the cell colony formation capacity after treatment with indicated compounds in CAL-51-sh53BP1 cells and their control counterparts (**d**) or in MCF-7-sh53BP1 cells and control-transfected cells (**e**). Cells were treated with 0, 5, 10 μM Olaparib with or without 10 μM KU55933 for 10 days. Up, representative pictures; bottom, quantitative data. All experiments were performed at least three times and data were statistically analyzed by two-tail t-test.*p < 0.05, **p < 0.01, ***p < 0.001. Error bars indicate S.E.M

The *53BP1* gene was stably knocked-down by transfecting CAL-51 and MCF-7 cell lines with sh53BP1 lentivirus particles. Particles were generated using two siRNAs to ensure efficient knock-down. Stable transfectants, named MCF-7-sh53BP1 and CAL-51- sh53BP1, were selected in the presence of puromycin for 10 days and maintained as polyclonal populations. Both transfected cell lines showed much lower 53BP1 expression than the control-transfected lines (Fig. 4a). Both transfected lines were also significantly less sensitive than control-transfected lines to 10 μM Olaparib, 10 μM KU55933 or both (Fig. 4b-c). Specifically, following combination treatment, proliferation inhibition rates were 20.3 % for MCF-7-sh53BP1 cells, 29.8 % for MCF-7-ctrl cells, 46.5 % for CAL-51-sh53BP1 and 57.4 % for CAL-51-ctrl cells (Additional file 1: Figure S2). Similar results were obtained in the colony formation assay (Fig. 4d-e). Taken together, our results suggest that 53BP1 deficiency renders ATM-inhibited cells less sensitive to Olaparib.

### Down-regulation of 53BP1 partially restored HR

**Fig. 5:**
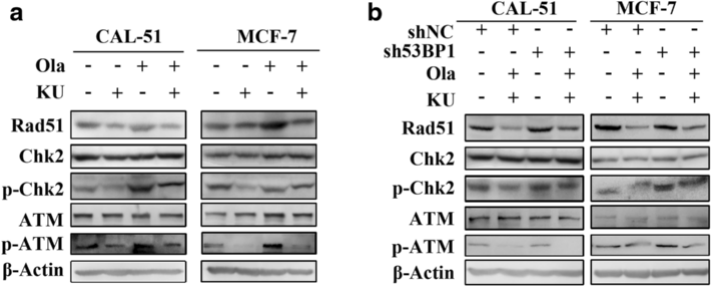
KU55933 reduced Olaparib induced ATM-Chk2 pathway activation and Rad51 up-regulation while knockdown 53BP1 supressed these effects. **a**, Western blot assay showed the protein level of Rad51, Chk2, phospho-Chk2 (Thr68), ATM and phosphor-ATM (Ser1981) in CAL-51 and MCF-7 cells after treatment with 10 μM Olaparib, 10 μM KU55933 and their combination respectively for 48 h. **b**, Western Blot assay showed the protein level of Rad51, Chk2, phospho-Chk2 (Thr68), ATM and phosphor-ATM (Ser1981) in CAL-51-sh53BP1, MCF-7-sh53BP1 cells and their control-transfected cells after synergistic treatment with 10 μM Olaparib and 10 μM KU55933. All experiments were performed at least three times

It is well accepted that ATM, Chk2 and Rad51 are critical regulators of HR DNA repair pathway. We sought to investigate the protein levels of these factors under the treatment with Olaparib, KU55933 or both. As is shown in Fig. 5a, Olaparib induced ATM phosphorylation and activated its downstream effector Chk2, and subsequently up-regulated Rad51. Adding KU55933 inhibited the activation of ATM and Chk2 and down-regulated Rad51. Taken together, these observations suggest that Olaparib activated the HR pathway and adding KU55933 inhibited it.

Previous studies have reported that knockdown of 53BP1 in BRCA1 deficient cells partially restored HR activity [22, 23]. Interestingly, we found that knock-down of 53BP1 also restored HR activity in ATM deficient cells. Higher levels of phospho-ATM and Chk2 and up-regulated RAD51 were observed in 53BP1 knock-down cells than their control counterparts when treated with Olaparib and KU55933 combined (Fig. 5b).

### Phospho-ATM and 53BP1 expression and overall-survival in triple-negative breast cancer

**Fig. 6:**
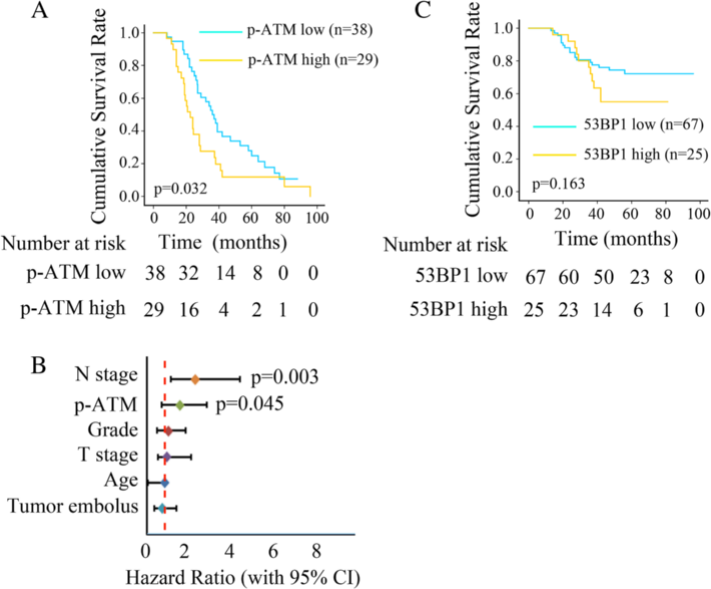
Association between phosphor-ATM or 53BP1 and overall survival in triple negative breast cancer (TNBC) samples A, Kaplan-Meier survival analysis of early TNBC patients stratified by phosphor-ATM expression level. B, Different factors (including N stage, phosphor-ATM level, grade, T stage, age and LVSI) were analyzed for their association with patient survival using Cox regression multivariate model. The hazard ratio and 95 % CI are plotted for each factor. C, Kaplan-Meier survival curves of early TNBC patients stratified by 53BP1 expression level

Our experiments link levels of phospho-ATM and 53BP1 to Olaparib sensitivity of breast cancer cell lines. Further, we examined whether these levels might correlate with survival of triple-negative breast cancer patients. Immunohistochemistry of FFPE tumor samples from 73 patients with early triple-negative breast cancer identified low expression levels of phospho-ATM in 44 (60.3 %) and high levels in 29 (39.7 %). Overall survival was significantly better in patients with low levels of phospho-ATM (Fig. 6a). After taking into account various clinical characteristics of the patients (Additional file 1: Table S2), and adjusting for age, tumor size, grade, number of metastatic lymph nodes and lymphovascular invasion, multivariate analysis identified phospho-ATM level as an independent predictor of overall survival (HR 1.83, 95%CI 1.063–3.164, *P* = 0.029; Fig. 6b).

Immunohistochemistry of FFPE tumor samples from another cohort of 92 patients with triple-negative breast cancer identified 67 (72.8 %) as negative for 53BP1. Overall survival tended to be better among these patients than among patients positive for 53BP1, but the difference did not achieve significance (Fig. 6c). The clinicopathological characteristics of this cohort are shown in Additional file 1: Table S3.)

## Discussion

PARP inhibitors have been studied most extensively in HSOC and triple-negative breast cancer, and have proven particularly effective against cancers associated with *BRCA1* or *BRCA2* mutations [28]. A phase II study in which gemcitabine and carboplatin were used with PARP inhibitor iniparib as neoadjuvant therapy in patients with triple-negative breast cancer reported a pathologic complete response rate of 56 % in cancer related to BRCA1/2 mutations and 33 % in cancer associated with wild-type BRCA1/2 [29]. In addition to BRCA1/2 mutations, perhaps as many as 35 % of patients with HSOC and triple-negative breast cancer may have other HR pathway defects, such as methylation-induced silencing of BRCA1/2, mutations in other DNA repair genes, or activation of HR inhibitors. This has led to the concept of “BRCAness” [30, 31], highlighting the importance of identifying biomarkers to detect HR defects, which may help predict types of cancer likely to respond to PARP inhibitors.

ATM plays a pivotal role in the cellular DNA damage response and is essential for maintaining genome stability. Low levels of ATM are associated with higher sensitivity to PARP inhibitors in several cancer cell lines [32]. ATM–deficient mantle-cell lymphoma and gastric cancer cells respond better to Olaparib than the ATM-proficient cells [19]. In a phase II clinical trial, treating gastric cancer patients with Olaparib and paclitaxel led to a greater increase in overall survival among the subgroup of patients with low ATM expression (HR 0.35, 80%CI 0.22 to 0.56, *P* < 0.002) than among the overall population (HR 0.56, 80%CI 0.41 to 0.75, *P* = 0.005) [33]. In the present study, using KU55933 to reduce the level of phospho-ATM sensitized breast cancer cell lines to Olaparib. This result raised the possibility that phospho-ATM level may predict sensitivity to PARP inhibitor in breast cancer. To test this, we looked for an association between survival of patients with early triple negative breast cancer and phospho-ATM expression in their tumor tissue. Using a cut-off of 50 % of tumor cells showing positive nuclear staining, we obtained separable overall survival curves f or patients above and below the cut-off. Moreover, multivariate analysis identified phospho-ATM level as an independent prognostic factor. Our results justify future clinical work to validate phospho-ATM level in breast cancer tissue as a predictor of PARP inhibitor sensitivity.

Our results indicate that PARP and ATM inhibition interact synergistically, reflecting the fact that both protein targets are involved in DNA damage repair and therefore interact synthetically. The effect of combination therapy was greater in triple-negative breast cancer cells than in non-triple-negative breast cancer lines. This is not surprising given that the most frequent loss-of-function and gain-of-function alterations in triple-negative breast cancer involve genes associated with DNA damage repair [34, 35]. Treating this type of breast cancer remains a major challenge because it is a highly heterogeneous disease [36] and no targeted therapy has been approved to date. Our results suggest the potential of combination treatment using PARP and ATM inhibitors, which should be validated and further examined in clinical trials.

In our study, 53BP1 down-regulation rendered ATM-deficient cells less sensitive to PARP inhibitor. This is consistent with a previous study showing that 53BP1 loss confers PARP inhibitor resistance in BRCA1-deficient tumor cells [22]. Our results further show that 53BP1 knock-down increased RAD51 focus formation, indicating partially restored HR. This is consistent with previous studies that loss of 53BP1 partially restores the impaired HR in BRCA1-deficient cells [22]. Given its link with HR, 53BP1 should be evaluated as a potential biomarker for identifying which patients will likely benefit most from the combination of ATM And PARP inhibition. A better Olaparib efficacy in gastric cancer patients with low ATM expression has been demonstrated in a phase II study, and 75 % of gastric carcinoma cases are reported to be 53BP1-positive34. In contrast, only 27.2 % of patients with triple-negative breast cancer in our cohort were 53BP1-positive, similar to the 30 % reported in a previous study [22] and lower than the 55 % reported for ER-positive breast cancer tissue.

## Conclusion

Overall, in our present study we confirmed that inhibiting ATM increased cytotoxicity of PARP inhibitor in breast cancer cell lines, and demonstrated for the first time that depleting 53BP1 reduced this cytotoxicity. We found that ATM inhibition abrogated homologous recombination induced by PARP inhibitor, and down-regulating 53BP1 partially reversed this effect. Further, overall survival was significantly better in triple-negative breast cancer patients with lower levels of phospho-ATM and tended to be better in patients with negative 53BP1. These findings indicated that 53BP1 might be a predictor of PARP inhibitor resistance in patients with ATM-deficient tumors.

## Additional file

Additional file 1:
**Figure S1.** Olaparib inhibited breast cancer cells proliferation and KU55933 enhanced this effect. A ~ D, MTS assay was used to determine cell viability of TNBC cell lines MDA-MB-231 (A), MDA-MB-468 (B) and non-TNBC cell lines T-47D (C), SK-BR-3 (D) under different concentration of compounds treatments (0, 1, 2.5, 5, 10 μM Olaparib with or without 10 μM KU55933, respectively) at 37 °C for 48 h. * means that there is statistical difference between the groups of Olaparib treatment with or without KU55933 at each indicating concentration. All experiments were performed at least three times and data were statistically analyzed by two-tail t-test.**p* < 0.05, ***p* < 0.01, ****p* < 0.001. Error bars indicate S.E.M. **Figure S2.** The inhibition effect of Olaparib treatment or combined with KU-55933 in breast cancer cells. MTS assays were performed to detecte the cell viability after treatment with indicated compounds for 48 h in CAL-51-sh53BP1 cells and their control counterparts or in MCF-7-sh53BP1 cells and control-transfected cells. Inhibition rate was calculated and normalized to the 0 μM Olaparib (DMSO) treatment group. The colors indicated the inhibition effect, blue, the stronger color means less inhibition; red, the stronger color means greater inhibition. **Table S1.** ER, PR, HER2, BRCA1 and BRCA2 status of selected cell lines. **Table S2.** Clinical characteristics of the cohort tested for pATM expression. **Table S3.** Clinical characteristics of the cohort tested for 53BP1 expression. (DOCX 819 kb)

## Abbreviations

PARP: Poly (ADP-ribose) polymerase
HSOC: High-grade serous ovarian cancer
PFS: Progression-free survival
DSB: Double-stranded DNA breaks
BER: Base excision repair
SSB: Single-stranded DNA breaks
HR: Homologous recombination
NHEJ: Non-homologous DNA end-joining
siRNA: Short interfering RNA
ATM: Ataxia-telangiectasia mutated
PIKK: Phosphatidyl-inositol kinase-like kinase
