# Erratum to: 53BP1 depletion causes PARP inhibitor resistance in ATM-deficient breast cancer cells

**DOI:** 10.1186/s12885-016-2970-1

**Published:** 2016-11-30

**Authors:** Ruoxi Hong, Fei Ma, Weimin Zhang, Xiying Yu, Qing Li, Yang Luo, Changjun Zhu, Binghe Xu, Wei Jiang

**Affiliations:** 1 Department of Medical Oncology, Cancer Institute and Hospital, Peking Union Medical College and Chinese Academy of Medical Sciences, Beijing, China; 2 Department of Medical Oncology, Sun Yat-sen University Cancer Center, Guangzhou, China; 3 State Key Laboratory of Molecular Oncology, Cancer Institute and Hospital, Chinese Academy of Medical Sciences and Peking Union Medical College, Beijing, China; 4 College of Life Science/Tianjin Key Laboratory of Cyto-Genetical and Molecular Regulation, Tianjin Normal University, Tianjin, 300387 China

## Erratum

After the publication of the article [1], the authors realised that the corresponding authors’ order need to be changed. Details as follows:

Correct corresponding author order: Binghe Xu^1*^ and Wei Jiang^3*^

Correct correspondence: xubinghe@medmail.com.cn; wjiang6138@cicams.ac.cn

Please note that the above author list and corresponding emails have been updated.
