# Supplementary material for: 53BP1 depletion causes PARP inhibitor resistance in ATM-deficient breast cancer cells
Source: BMC Cancer. 2016 Sep 9;16(1):725. doi: 10.1186/s12885-016-2754-7 (PMC5017014; doi:10.1186/s12885-016-2754-7)
Supplement: Additional file 1: — Figure S1. Olaparib inhibited breast cancer cells proliferation and KU55933 enhanced this effect. A ~ D, MTS assay was used to determine cell viability of TNBC cell lines MDA-MB-231 (A), MDA-MB-468 (B) and non-TNBC cell lines T-47D (C), SK-BR-3 (D) under different concentration of compounds treatments (0, 1, 2.5, 5, 10 μM Olaparib with or without 10 μM KU55933, respectively) at 37 °C for 48 h. * means that there is statistical difference between the groups of Olaparib treatment with or without KU55933 at each indicating concentration. All experiments were performed at least three times and data were statistically analyzed by two-tail t-test.*p < 0.05, **p < 0.01, ***p < 0.001. Error bars indicate S.E.M. Figure S2. The inhibition effect of Olaparib treatment or combined with KU-55933 in breast cancer cells. MTS assays were performed to detecte the cell viability after treatment with indicated compounds for 48 h in CAL-51-sh53BP1 cells and their control counterparts or in MCF-7-sh53BP1 cells and control-transfected cells. Inhibition rate was calculated and normalized to the 0 μM Olaparib (DMSO) treatment group. The colors indicated the inhibition effect, blue, the stronger color means less inhibition; red, the stronger color means greater inhibition. Table S1. ER, PR, HER2, BRCA1 and BRCA2 status of selected cell lines. Table S2. Clinical characteristics of the cohort tested for pATM expression. Table S3. Clinical characteristics of the cohort tested for 53BP1 expression. (DOCX 819 kb) [file 12885_2016_2754_MOESM1_ESM.docx]

**Supplementary Figures**

**Supplementary Figure S1** **Olaparib inhibited breast cancer cells proliferation and KU55933 enhanced this effect.** A~D, MTS assay was used to determine cell viability of TNBC cell lines MDA-MB-231 (A), MDA-MB-468 (B) and non-TNBC cell lines T-47D (C), SK-BR-3 (D) under different concentration of compounds treatments (0, 1, 2.5, 5, 10 μM Olaparib with or without 10 μM KU55933, respectively) at 37°C for 48h. * means that there is statistical difference between the groups of Olaparib treatment with or without KU55933 at each indicating concentration. All experiments were performed at least three times and data were statistically analyzed by two-tail t-test.*p<0.05, **p<0.01, ***p<0.001. Error bars indicate S.E.M

**Supplementary Figure S2 The inhibition effect of Olaparib treatment or combined with KU-55933 in breast cancer cells.** MTS assays were performed to detecte the cell viability after treatment with indicated compounds for 48 h in CAL-51-sh53BP1 cells and their control counterparts or in MCF-7-sh53BP1 cells and control-transfected cells. Inhibition rate was calculated and normalized to the 0 μM Olaparib (DMSO) treatment group. The colors indicated the inhibition effect, blue, the stronger color means less inhibition; red, the stronger color means greater inhibition.

**
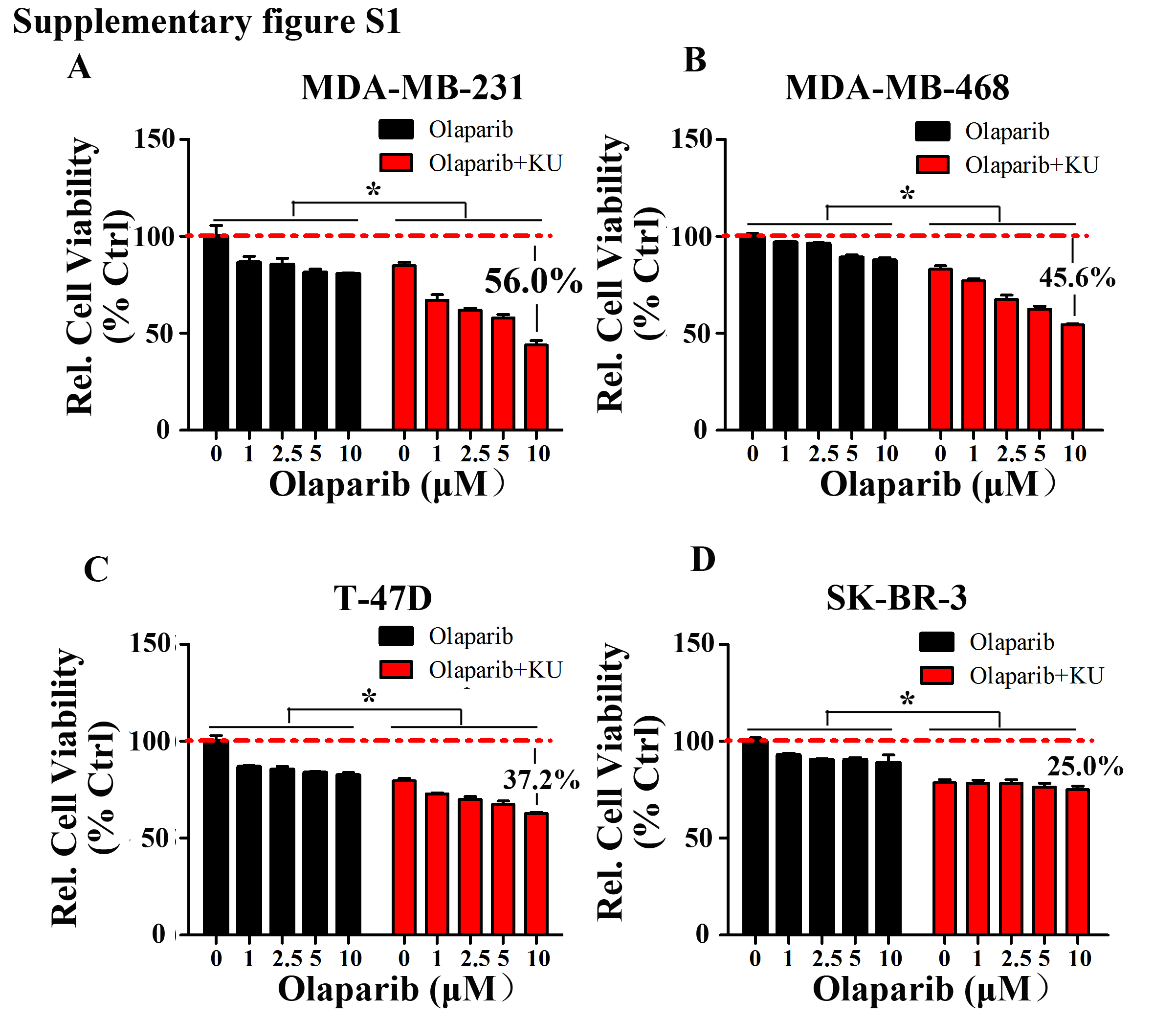
**

**
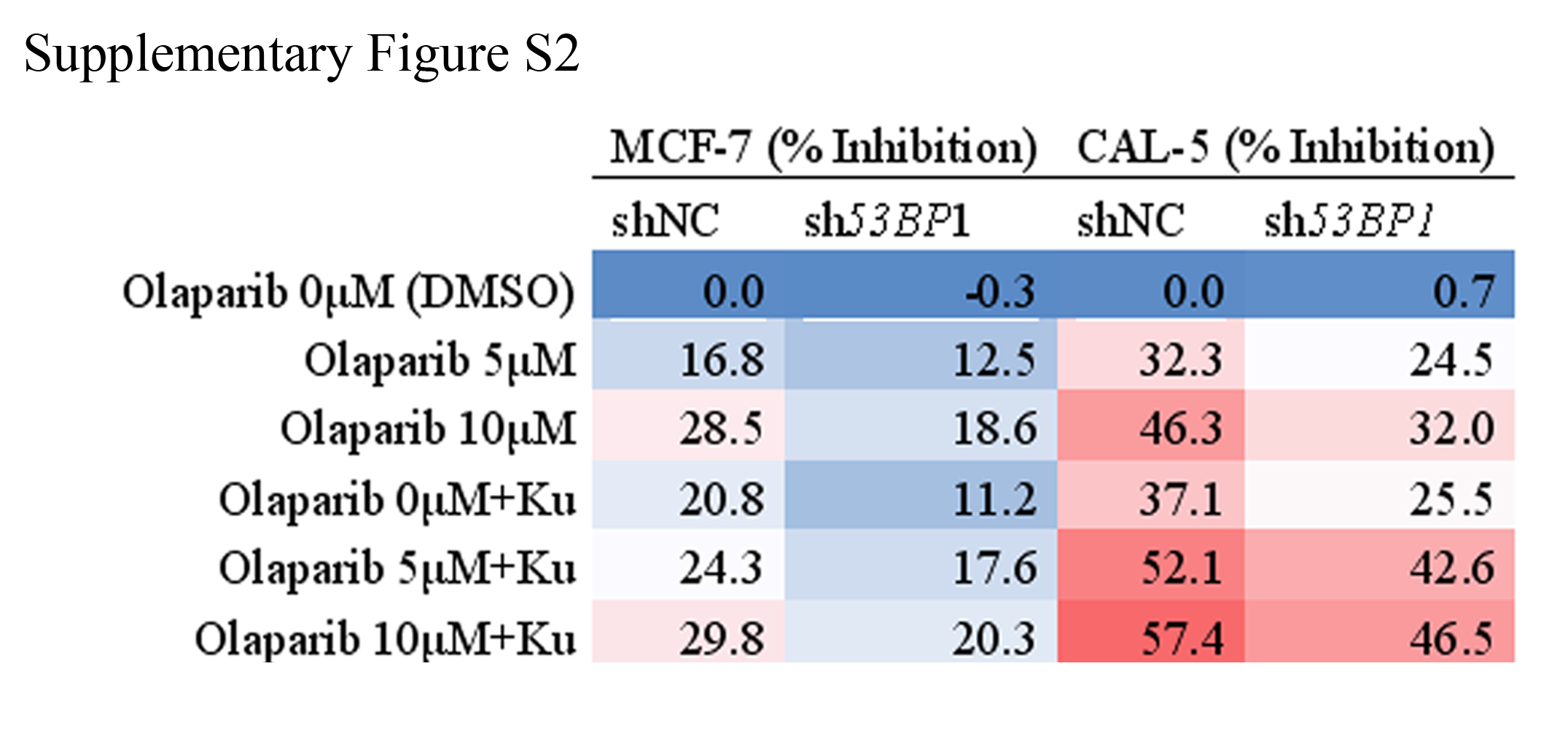
**

**Supplementary Tables**

**Supplementary Table S1.** ER, PR, HER2, BRCA1 and BRCA2 status of selected cell lines

|  | ER | PR | ERBB2 | BRCA1/2 |
| --- | --- | --- | --- | --- |
| CAL-51 | - | - | - | WT |
| 231 | - | - | - | WT |
| 468 | - | - | - | WT |
| MCF-7 | + | + | - | WT |
| T47D | + | + | - | WT |
| SKBR3 | - | - | + | WT |

**Supplementary Table S2.** Clinical characteristics of the cohort tested for pATM expression

|  | Total (n=73) | High pATM  expression (n=29) | Low pATM expression (n=44) | P value |
| --- | --- | --- | --- | --- |
| Age (mean±SD) | 49±11.5 | 50±14.3 | 48±10.9 | 0.618 |
| Histology |  |  |  | 1.000 |
| IDC | 70 (95.9%) | 28 (96.6%) | 42 (95.4) |  |
| others | 3 (4.1%) | 1 (3.4%) | 2 (4.6%) |  |
| Poor differentiation |  |  |  | 0.665 |
| No | 35 (47.9%) | 13 (44.8%) | 22 (50%) |  |
| Yes | 38 (52.1%) | 16 (55.2%) | 22 (50%) |  |
| Tumor size ≥5cm |  |  |  | 0.227 |
| Yes | 15 (20.5%) | 8 (27.6%) | 7 (15.9%) |  |
| No | 58 (79.5%) | 21 (72.4%) | 37 (84.1%) |  |
| LN number ≥4 |  |  |  | 0.164 |
| Yes | 38 (52.1%) | 18 (62.1%) | 20 (45.5%) |  |
| No | 35 (47.9%) | 11 (37.9%) | 24 (54.5%) |  |
| Lymphangio-invasion |  |  |  | 0.977 |
| Yes | 20 (27.4%) | 8 (27.6%) | 12 (27.3%) |  |
| No | 53 (72.6%) | 21 (72.4%) | 32 (72.7%) |  |

**Supplementary Table S3.** Clinical characteristics of the cohort tested for 53BP1 expression

|  | Total (n=92) | 53BP1 positive (n=25) | 53BP1 negative (n=67) | P value |
| --- | --- | --- | --- | --- |
| Age (mean±SD) | 50±11.6 | 52±12.0 | 49±11.5 |  |
| Histology |  |  |  | 0.297 |
| IDC | 88 (95.7%) | 23 (92.0%) | 65 (97.0%) |  |
| others | 4 (4.3%) | 2 (8.0%) | 2 (3.0%) |  |
| Poor differentiation |  |  |  | 0.154 |
| No | 39 (42.4%) | 14 (56.0%) | 25 (37.3%) |  |
| Yes | 53 (57.6%) | 11 (44.0%) | 42 (62.7%) |  |
| Tumor size ≥5cm |  |  |  | 1.000 |
| Yes | 13 (14.1%) | 3 (12.0%) | 10 (14.9%) |  |
| No | 79 (85.9%) | 22 (88.0%) | 57 (85.1%) |  |
| LN number ≥4 |  |  |  | 1.000 |
| Yes | 29 (31.5%) | 8 (32.0%) | 21 (31.3%) |  |
| No | 63 (68.5%) | 17 (68.0%) | 46 (68.7%) |  |
| Lymphangio-invasion |  |  |  | 0.811 |
| Yes | 55 (59.8%) | 14 (56.0%) | 41 (61.2%) |  |
| No | 37 (40.2%) | 11 (44.0%) | 26 (38.8%) |  |
